# Practical Applications of Bioelectric Stimulation

**DOI:** 10.1089/bioe.2019.29011.rnu

**Published:** 2019-12-12

**Authors:** Richard Nuccitelli

**Affiliations:** Department of Biology, Pulse Biosciences, Hayward, California.

It has been a great honor to be a guest editor for this special issue on the *Practical Uses of Bioelectric Stimulation*. Here you will find six examples of the use of imposed or modified electric fields to greatly influence tissue functions. This is only a very small sampling of the thousands of practical uses of bioelectricity today, and many more examples of this subtopic will appear regularly in this journal.

The discipline of bioelectricity encompasses both endogenous ionic currents and electric fields used by living organisms in their daily functions as well as the use of imposed electric fields to influence or modify these functions. The endogenous ion channels and currents associated with cancer cells was highlighted in the September issue that contained eight articles focused on the bioelectricity of cancer and covered many characteristics of cancer cells that are intimately associated with bioelectricity, including the control of metastasis, the localization of specific ion channels, and the influence of extracellular K^+^ on the immune system. In contrast, this issue of *Bioelectricity* focuses on the use of imposed electric fields to modify cell and organism functions.

The common theme of four of these bioelectricity applications is the use of pulsed electric fields to influence cell permeability. The first article is a broad review of the past 5 years of electrochemotherapy, the use of microsecond-domain electric fields that trigger transient or reversible permeabilization to locally enhance the uptake of drugs that kill dividing cancer cells. This approach has been used extensively in Europe but is not yet approved for use in the United States. In human oncology electrochemotherapy is fully recognized as a local therapy for cutaneous tumors and metastases and its effectiveness is also being explored in combination with immunomodulatory drugs.

The second article reviews irreversible electroporation (IRE), the use of larger pulsed electric fields in the microsecond domain that generates permanent pores in the plasma membrane resulting in the loss of homeostatic equilibrium leading to necrosis within minutes to hours. This technique has been approved for human use in both the United States and Europe for soft tissue ablation. It has established a clinical niche as an alternative to thermal ablation for the eradication of unresectable tumors, particularly those near critical vascular structures. IRE clinical data have shown promise in treating tumors in the prostate, pancreas, and liver.

The third article describes the use of much shorter electrical pulses to treat skin lesions called Nano-Pulse Stimulation. This is a nonthermal technology that has been used to eliminate several types of skin lesions in clinical trials approved as nonsignificant risk trials supervised by IRBs and has not yet been approved for human use. These pulses generate reversible nanopores in all membranes exposed to them that are much smaller than the pores generated by the longer microsecond pulses. In addition, they have been shown to penetrate into cells and permeabilize the organelles as well as the plasma membrane resulting in the initiation of regulated cell death when a sufficient number of pulses are applied.

The fourth article describes a very new application of Nano-Pulse Stimulation, cardiac defibrillation. The standard method for defibrillation uses much longer millisecond pulses that often cause long-term damage to the heart. Nano-Pulse Stimulation requires only about 12% as much energy to defibrillate so will probably result in less tissue damage.

The fifth article is an exception to the increased permeability theme and instead provides a review of tissue inflammation and regeneration responses to imposed sinusoidal magnetic fields. In addition, some new data are presented showing that 5 and 15 Hz sinusoidal magnetic fields can induce very weak electric fields that can modulate inflammation and tissue regeneration. The mechanisms by which such low sinusoidal fields affect inflammation are likely to be very different from those used by much higher amplitude pulsed fields. We have much to learn about the targets of and mechanisms used by these weak oscillating electromagnetic fields.

The final article in this special issue utilizes a light-activated ion channel to modify the membrane potential of brain tissue and reverse the abnormal brain and eye morphologies created by ethanol exposure. This is a striking demonstration of the critical role of membrane potential in the development of the brain that was not possible before optogenetics came on the scene. It is an amazing example of the signaling power of bioelectricity brought to light through the use of technological advances.

